# ASO Author Reflections: Internal Mammary Sentinel Lymph Node Biopsy—Time for the Back of Internal Mammary Staging?

**DOI:** 10.1245/s10434-019-07838-2

**Published:** 2019-11-06

**Authors:** Peng-Fei Qiu, Yong-Sheng Wang

**Affiliations:** Breast Cancer Center, Shandong Cancer Hospital and Institute, Shandong First Medical University and Shandong Academy of Medical Sciences, Jinan, Shandong China

## Past

Axillary lymph node (ALN) and internal mammary lymph node (IMLN) are the first-echelon nodal drainage site of breast cancer, yet the primary interest of breast surgeons has been focused on ALN while IMLN were largely ignored, since data from extended radical mastectomy in the 1960s showed no advantages in survival with the IMLN dissection.^1^ With the prominent survival benefit of IMLN radiation, more attention should be paid again to the staging and management of IMLN. Although the internal mammary sentinel lymph node biopsy (IM-SLNB) is a minimally invasive IMLN staging technique, its routine performance remains controversial for the following reasons.^2^ First, the internal mammary sentinel lymph node (IMSLN) were only visualized in a small proportion of patients (15%) with traditional radiotracer injection technique, which has been the restriction for IM-SLNB. Second, the IM-SLNB was only performed in cN0 patients, which led to the low IMLN metastasis rate (8–15%) and little clinical relevance.

## Present

One of the primary goal of breast surgery nowadays is nodal staging, which will not be completed without both axillary and IM-SLNB. We tried injecting radiotracer with modified technique (periareolar intraparenchymal, high volume, and ultrasound guidance) in cN0 patients and got a high IMSLN visualization rate of 71.1%, which laid a technical feasibility for further study and clinical application.^3^ In this study, we performed IM-SLNB in cN + patients and reconfirmed the IMSLN visualization rate. The IMSLN metastasis rate of patients who received initial surgery and neoadjuvant systemic therapy was 39.8% and 13.3%, respectively. Patients who received IM-SLNB will have more accurate nodal staging, which might potentially affect the therapeutic strategies, including individual IMLN irradiation.^4^ As a minimally invasive staging technique, we suggest that IM-SLNB should be routinely performed during mastectomy, especially in cN + patients, and performed selectively during lumpectomy in high IMLN metastatic risk patients (positive-ALN and/or medial tumor), as an additional 3-cm skin incision might be required.^5^

## Future

High visualization rate and low false-negative rate are prerequisites for the widespread of IM-SLNB. The question arises as to whether IMSLN detected with the modified technique should be considered as the “true” IMSLN. We are conducting two prospective multicenter studies: CBCSG026 trial (NCT03541278) was designed to verify the repeatability of this high IMSLN visualization rate in patients with both ALN negative and positive breast cancer (a minimum of 350 patients for enrollment); the CBCSG027 trial (NCT03024463) of IM-SLNB followed by the 1^st^ to 3rd intercostal IMLN dissection was designed to verify the IM-SLNB accuracy in ALN-positive patients (at least 40 patients with positive IMLN required). Seven centers have enrolled more than three quarters patients in both trials before April 30. The overall IMSLN visualization rate is 68.2% with IM-SLNB success rate of 94.9%. The overall IMLN positive rate is 42.0% with a false negative rate of 2.9% (data not shown). We hope these two trials could provide clinical practice-changing evidence.
